# Worcester Infirmary

**Published:** 1829-04-01

**Authors:** 


### WORCESTER INFIRMARY.

A case of dislocation into the ischiatic notch occurred at the above institution, and was easily reduced in less than ten minutes. The injury happened from a quantity of gravel falling on the patient whilst at work in a pit. Besides the dislocated hip, there was compound fracture of the other leg. The wound united by the first intention, and the man in six weeks was sent out cured, a circumstance which, we venture to say, would not have taken place in a London hospital.

II. ANEURISM OF THE POSTERIOR TIBIAL ARTERY.—A labouring man, ætatis 48, was admitted the 17th of July, 1825, and placed under the care of Mr. Shepherd. He complained of pain in the right leg, where a tumour appeared beneath the gastrocnemius, hard, diffused, painful on pressure, and pulsating strongly. Pressure on the femoral artery arrested the pulsation, and violent pain was produced on allowing the blood suddenly to re-enter the tumour. No pulse could be felt in the posterior tibial, as it turns round the inner ankle, but that of the anterior tibial was distinguished. The pulse at the wrist was remarkably strong and 90 in the minute—tongue furred and dry—appetite bad—head-ache—no cough. The disease had commenced with pain and stiffness three weeks before, followed shortly afterwards by the swelling and pulsation, and numbness extending down the leg to the sole of the foot. Sixteen ounces of blood were taken from the arm, the bowels freely opened, and cold lead lotion applied to the leg. On the 20th, the femoral artery was tied with a single ligature just where it is crossed by the sartorius, when all pulsation ceased in the tumour, though the pain still continued. In the evening, no pulsation was felt in the anterior tibial artery, but the temperature of the limb was higher in the other. On the 22d, the wound had almost united by the first intention, and the tumour was much diminished in size. The pain in the tumour was a great deal less, there was none whatever in the wound, but a pricking sensation was experienced in the foot.

On the 5th of August, a small abscess had formed near the upper end of the wound, which burst in the night, and on the 7th, the ligature on the vessel came away. On the 31st, the leg was reduced to its natural size, and the health was much improved; but pain was occasionally felt in the foot, and a sinuous opening at the upper part of the wound was still discharging a small quantity of pus. On the morning of the 31st, the poultice on the sinus was found to be tinged with blood, and the margins were hardened, tender, and inflamed. In the evening, the reporter was summoned to the patient on account of arterial hæmorrhage from the wound issuing in a stream as large as a crow-quill. Pressure on the artery above restrained it, only to recur when the pressure was removed, and requiring, therefore, its constant adoption.

"By midnight, a large quantity of blood had become diffused in the cellular membrane of the thigh, forming an aneurism, and by the morning it had increased to an immense size, extending upwards nearly to Poupart's ligament, and pulsating violently. The hæmorrhage through the night could with difficulty be restrained by keeping up a firm and constant pressure on the artery above, and over the wound in the thigh. Did not complain of much pain; his tongue was furred and dry; skin hot; pulse 74, and hard."

Early the following morning the external iliac artery was immediately and with ease secured by Mr. Shepherd. No bad symptom followed of any kind; a quantity of thick coagulum mixed with pus was discharged from the original wound in the thigh, which did not, however, suppurate to any extent; the ligature came away on the 27th day; and October 20th, the limb having regained its natural size, and nothing except a slight stiffness remaining, the patient was dismissed the infirmary cured. We should mention that four days after the second operation the abdominal aorta was seen and felt pulsating strongly, and on the 11th, a deep pulsation was felt in the femoral, below Poupart's ligament.

The above is a valuable and interesting case; valuable, as exhibiting a sample of judicious surgical practice; interesting, both from the phenomena it presented, and the final and fortunate result. The operation in the thigh, the ligature applied to the femoral artery, cured the aneurism in the calf of the leg, by stemming, not stopping the current of blood. So far the case is satisfactory enough, but why should secondary hæmorrhage take place, at so late a period as twenty-four days after the separation of the ligature from the vessel? Mr. Hodgson, in his work on Diseases of the Arteries, without exception the most valuable that ever was published on the subject, observes, that secondary hæmorrhage occurs at two periods after the application of a ligature to an artery:—either within a few hours after the operation, or between the sixth and the thirtieth day, when the ligature may be expected to be detached from the vessel. It is obvious that this case is different from both, for the hæmorrhage occurred, not as the immediate consequence of the operation, nor yet of the separation of the ligature, but nearly a month after the latter had taken place. We have witnessed a nearly similar case. It was that of a patient with popliteal aneurism whose femoral artery was tied in the thigh. In twenty-one days the ligature came away, and a *fortnight* after that, uncontrollable secondary hæmorrhage occurred. The common femoral was then secured, and *thirteen* days exactly, after the separation of this second ligature, secondary, and ultimately fatal hæmorrhage ensued.

We repeat then our question, what is the cause of secondary hæmorrhage at this late period? Whether does it arise from insufficient coagulum, weak adhesions of the divided coats, or ulceration, extending from without? for in all of the cases, sinuses communicating with the wound remained. We do not know ourselves, nor have we found any one who did.

III. Tumour on the Orbit—Abscess of the Cerebrum.—A. B. ætatis 45, a labouring man, entered the infirmary, at the latter end of February, 1824, with pain and inflammation of the left eye, and a fistulous opening beneath the superciliary ridge, admitting a probe to the back part of the orbit. A fortnight after his admission, he was seized with shivering, nausea, quick pulse, somnolency alternating with restlessness, hot and dry skin, flushed face. These symptoms subsided; but suddenly, on the 30th of March, he became insensible, and the muscles on the *right* side of the body were paralysed. There was stertorous breathing, immoveable contraction of the pupil to a point, slow pulse. Some white curdy matter escaped from the fistulous opening above the eye, and trepanning of the frontal bone above the orbit was proposed but not done. Leeches were applied to the head—blood drawn from the temporal artery, and the bowels with difficulty opened by strong purges and clysters of turpentine. All was in vain, for in five days after the appearance of the apoplectic symptoms, he died.

"*Examination of the body*, 24 *hours after death.*—A tumour, white and cheesy, of the size of a large walnut, lay above the left Orbit, about the middle of the Superciliary Ridge. It appeared to have been formed between the tables of the Frontal Bone, which it had separated from each other, producing absorption of the Cancellar Structure: also of the Inner Table, and of the Orbitary Plate, so that the Tumour was in contact with the Eye-ball beneath, and the Dura Mater behind. The Dura Mater was adherent to the Tumour on one side, and to the Pia Mater on the other, and at the place of adhesion was blackened for the space of a shilling. Immediately behind the Tumour was an Abscess, occupying the whole anterior Lobe of the left hemisphere of the Brain, and containing three ounces of fetid pus. The Medullary substance of the Brain, around the Abscess, was softened, and had a yellow tint. The vessels of the Brain were turgid with blood, and there were two ounces of serum in the Lateral Ventricles."

What extensive mischief in the brain had occurred, and yet how insidious, how trifling the symptoms were! This is not uncommon in cases of abscess of the brain, and should teach the practitioner caution in diagnosis and prognosis. As the case turned out, it was well that trephining was not had recourse to.—*Midland Reporter.*

